# CircSND1/miR-182-5p Axis Promotes Proliferative and Invasive Abilities of Thyroid Cancer via Binding Targeting MET

**DOI:** 10.1155/2022/9175084

**Published:** 2022-05-30

**Authors:** Dongliang Wang, Shuilong Zhang, Dewei Li, Qiang Wang, Zhifu Xiao, Yuhang Zhang

**Affiliations:** Thyroid Surgery of Shanxi Provincial People's Hospital, Taiyuan, Shanxi 030012, China

## Abstract

**Objective:**

To monitor the impacts of circSND1 upon thyroid cancer (TC) tissues and cells and its mechanisms.

**Methods:**

Thiazole blue (MTT) was adopted to monitor the impacts of circSND1 upon the proliferative abilities of TPC-1 and SW1736 cells. 5-Bromodeoxyuridine (BrdU) combined with flow cytometry was adopted to monitor the impacts of circSND1 upon the DNA synthesis of TPC-1 and SW1736 cells. We adopted transwell experiment to examine the impacts of circSND1 on cell invasive abilities of TPC-1 and SW1736 cells. The mRNA quantitative levels of circSND1, miR-182-5p, and mesenchymal epidermal transformation factor (MET) in TC tissues were detected by qRT-PCR experiment. We also adopted luciferase assay to verify the targeting interaction between miR-182-5p and MET or miR-182-5p and circSND1.

**Results:**

CircSND1 mRNA and MET mRNA were upregulated in thyroid cancer tissues. MiR-182-5p quantification was attenuated in thyroid cancer tissues. Downregulation of circSND1 suppressed TC progression in vivo and in vitro. Furthermore, luciferase report assay uncovered that miR-182-5p was a direct binding target of circSND1 and MET was a direct binding target of miR-182-5p. Besides, circSND1 regulated MET expression and thyroid cancer cell function via binding miR-182-5p.

**Conclusion:**

Overexpression of circSND1 in TC tissues and cells facilitates TC tumorigenesis and metastasis via suppressing the quantitative level of miR-182-5p and inducing the upregulation of MET mRNA and protein expression, which expected to offer fresh clues for the administration of TC.

## 1. Introduction

Thyroid cancer (TC) is a malignant tumor growing in the thyroid gland, with a rapid growth rate in the past several years around the world [1]. In terms of the World Cancer Report in 2014, approximately two hundred and ninety-eight thousand individuals were newly diagnosed with TC and forty thousand individuals heading towards death [2]. In 2015, TC was already the sixth most malignant tumor among females and the most common tumor among females under the age of 30 [3]. Studies have shown that, in patients with local metastases of TC, the overall 10-year rate of survivors is 75%, while it is merely 21–40% for stage IV sufferers [4]. In addition, the recurrence rate of TC is very high, causing a decrease in the recovery rate and high mortality, which seriously endangers human health [5]. Currently, the most common treatment for TC is combination therapy of surgical removal and chemotherapy. It does not have satisfactory effects on resistant/undifferentiated/carcinoma yet, and there are still controversies in cancer recurrence and harm to the human body. Therefore, exploring the tumorigenesis mechanism of TC and finding the key molecules that affected the proliferative/invasive abilities of TC cells are an important approach for TC therapy.

Different from traditional linear RNA molecules, circular RNA (circRNA) is of the same category of RNAs that does not encode proteins and simultaneously forms a circus by covalent bonds. Unlike other noncoding RNAs, circRNAs lack a 5′-end cap and a 3′-end poly (A) tail structure, are expressed stably in numerous tissues and cells, and usually escaped the degradation of RNA enzymes. In 1976, researchers first discovered circRNA in plant viruses [6]. In recent years, researches on circRNA have shown a blowout pattern. A bulk of literature have uncovered that circRNAs play an increasingly importould like role in regulating process of more and more diseases, especially tumors, although their mechanisms have been not fully understood [7–10]. One of the most investigated functions of RNA is ceRNA/miRNA-sponge. CircRNA reduces the binding effect of miRNA to target mRNA by competing with related miRNAs, which also known as ceRNA mechanisms [11]. Many circRNAs have multiple miRNA binding sites and regulate their expression at the same time. These evolutionarily conserved binding sites can ensure the efficiency of binding to target miRNAs. Wei et al. [12] disclosed that CircCDYL is highly expressed in the plasma of liver cancer patients, and the mechanism is through the sponging of miR-892a/miR-328-3p. In addition, circRNA exerts their biological functions by regulating gene expression. Zhang et al. [13] found that ci-ankrd52 is abundant in the nucleus but is rarely enriched in microRNA target sites; however, it can directly regulate the expression of ankrd52 gene by combining with PolII transcribed from ankrd52 gene, which can be downregulated after ci-ankrd52 knockdown. CircRNA also binds to RNA binding protein. For example, Memczak et al. [14] and other studies have proved that human circRNA-ciRS-7 transcripts can intensively bind to AGO protein and form a large class of posttranscriptional regulatory factors. There are also countless circRNAs that mainly play regulatory functions through ceRNA mechanisms in thyroid cancer. For example, human circRNA 102002 accelerates TC metastasis via binding miR-488-3p [15]. Human circRNA 0011385 enhances TC progresses via sponging miR-361-3p [16]. CircSND1 was a novel RNA, and Bai et al. [17] uncovered that circSND1 intensified FUT6 quantification and the malignant behavior of carcinoma of uterine cervix through the ceRNA mechanisms.

However, the functions of circSND1 in TC tissues and cells have not yet been elucidated. Therefore, in current literature, we established a low-expression model of circSND1 in human thyroid cancer cell lines, mainly to explore the effects of circSND1 on TC progression and whether circSND1 can target miR-182-8p/MET axis.

## 2. Materials and Methods

### 2.1. Patient Tissue Samples

26 TC tissue samples and their corresponding adjacent tissues were collected from the Shanxi Provincial People's Hospital. Patients ranged in age from 19 to 76 years, with a median age of 49 years. According to the 2015 AJCC/UICC staging protocol for thyroid cancer, paracancer tissue >2 cm away from the tumor edge (no cancer cells were seen under the microscope) was selected as control. The patients had signed an informed consent form before the operation, and the patients and their families had clearly defined the purpose and significance of this study. This study has obtained approval from the ethics committee.

### 2.2. Cell Culture

Human TC cell lines (TPC-1, BCPAP, SW1736 cells) and human normal thyroid epithelial cell TEC cells were purchased from the Shanghai Life Science Research Resource Center of the Chinese Academy of Sciences. Inoculate cells in DMEM medium containing 10% fetal bovine serum (FBS), and place them in 37°C and 5% CO_2_ cultivation in an incubator.

### 2.3. Cell Transfection

TPC-1 and SW1736 cells in the logarithmic growth phase were seeded into a 6-well plate at a density of 1 × 10^5^ cells/well and cultured in a constant temperature incubator at 37°C and 5% CO_2_ for 24. Cells were transfected with sh-NC and sh-circSND1 using Lipofectamine 2000 reagent and continue to culture for 48 h. TPC-1 cells were transfected with sh-NC + inhibitor-NC, sh-circSND1 + inhibitor-NC, sh-NC + miR-182-5p inhibitor, or sh-circSND1 + miR-182-5p inhibitor using Lipofectamine 2000 reagent and continue to culture for 48 h.

### 2.4. RNA Extraction and qRT-PCR Assay

In terms of the TRIzol kit (Invitrogen) instructions, the total RNAs in thyroid tissue, BCPAP, TPC-1, and SW1736 cells were extracted. Follow the reverse-transcription kit (Applied Biosystems) instructions to synthesize cDNA using reverse transcriptase. Construct a PCR system according to the SYBR GREEN kit instructions. Use U6 or GAPDH as internal reference. circSND1-Forward: 5′-CGGGGTACCATGGCAACATCACAGAGCTCCTCCT-3′, circSND1-Reverse: 5′-CCGCTCGAGCCTGGGTGTTCTCCCCCTCCAGCCT-3′. The results are calculated using the 2^−ΔΔCt^ method.

### 2.5. MTT Assay to Examine Cell Viabilities

Transfected TPC-1 and SW1736 cells were, respectively, seeded into 96-well plates at a density of 2,000 cells/well, incubated in an incubator for 24 h. Subsequently, 20 *μ*g/ml MTT reagent was supplemented to each well, and the incubation was continued for another 4 h at 37°C. Finally, 150 *μ*l DMSO was supplemented to terminate the incubation, employing the microplate reader to read absorbance (OD) value of each well at 490 nm. Each experiment was repeated 3 times.

### 2.6. BrdU Staining

BrdU was added to cells in the critical logarithmic growth phase to a final concentration of 30 *μ*M and reaction for 30–60 minutes. Collect cells, resuspend each tube with 500 *μ*L washing solution and wash twice, and adjust the number of cells to 10^5^-10^6^/tube. 500 *μ*L fixative reagent was added to each tube to resuspend the cells and was fixed at 4° overnight. 500 *μ*L permeabilization solution was added to each tube to resuspend the cells and was incubated on ice for 2 minutes. The cells were resuspended in 300 *μ*L DNA denaturation working solution, incubated at 37°C for 30 minutes, and washed twice with 500 *μ*L washing solution. The cells were resuspended in 195 *μ*L staining buffer, supplemented 5 *μ*L (0.125 *μ*g) of PE-BrdU antibody per tube, and incubated at 4°C for 30 min in the dark. We used flow instrument to detect the intensity, 488 nm excitation wavelength, and 575 nm emission wavelength.

### 2.7. Transwell Invasion Assay

Diluted Matrigel gel with serum-free cell culture medium or PBS buffer at a ratio of 1 : 8 at 4°C, take 100 *μ*L, and apply it evenly on the surface the polycarbonate membrane surface of the chamber at 37°C for 0.5–1 h to polymerize it into a gel. We took treated TPC-1 and SW1736 cells in the logarithmic growth phase and washed them with PBS, then the cells were resuspended in serum-free medium, and the cell density was adjusted to 1–10 × 10^5^ cells/mL. 500–650 *μ*L of medium containing 5%–10% FBS or chemokines are generally added to lower chamber of 24-well plate. And then the transwell chamber is placed in the 24-well plate, and 100–200 *μ*L of the cell suspension is added to the upper chamber. And finally we put it in the incubator for 12–48 h. Take out the cell, aspirate the medium, and gently wipe the cells in Matrigel and the upper chamber with a cotton swab. Take a new 24-well plate and add 600 *μ*L of 4% paraformaldehyde, put it in the chamber, and fix it for 20–30 minutes. Subsequently, we discarded the fixative solution, stained it with 0.1%–0.2% crystal violet for 5–10 min, and washed it 3 times with PBS to remove the crystal violet that is not bound to the cells. After air-drying, select 5 fields of view under a high-power microscope to observe and count the cells.

### 2.8. Thyroid Cancer Tumor Xenograft In Vivo

The animal operations were agreed upon by the Animal Management Committee of the Shanxi Provincial People's Hospital. 6-week-old BALB/c athymic female nude mice (Mediclilon, Shanghai, China) were obtained for in vivo research. TPC-1 cells transfected with sh-NC (*n* = 6) and sh-circSND1 (*n* = 6) were diluted in 100 *μ*L medium and mixed fully and then injected subcutaneously into the dorsal skin of every nude mouse (5 × 10^6^ cells of each mouse). We used a digital caliper to measure tumor volume on the 8th, 16th, 24st, and 32th day after injection and calculated tumor volume. Nude mice were sacrificed, and the tumor mass was taken and weighed after all experiments were completed.

### 2.9. Dual-Luciferase Reporter Assay

After amplifying the MET or circSND1, it was cloned into the Renilla luciferase psiCHECK2 vector to construct a wild-type MET or circSND1 plasmid (wt-MET or wt-circSND1). The MET or circSND1 was mutated and cloned into the vector to construct a mutant MET or circSND1 plasmid (mut-MET or mut-circSND1). The Lipofectamine 2000 kit was used to cotransfect wt-MET and mut-MET or miR-NC and miR-182-5p mimics. The Lipofectamine 2000 kit was used to cotransfect wt-circSND1 and mut-circSND1 or miR-NC and miR-182-5p mimics. And the intracellular luciferase activity was measured 24 hours later. Each experiment was repeated 3 times.

### 2.10. Immunohistochemical Staining

Prepared paraffin sections were first dewaxed by xylene and fixed employing 4% paraformaldehyde. Then the endogenous catalase was then removed by soaking in a methanol solution containing 3% H_2_O_2_ for 10 min. Citric acid buffer was added and cooked intermittently in microwave oven for 3 min to expose the epitopes. Goat serum was supplemented for 30 min to seal some nonspecific sites. We took out the slides, dried the residual serum around the slide with absorbent paper, and, subsequently, adopted MET primary antibody (1 : 3000, Cell Signaling Technology) to cover sections for about 12 h at 4°C. Afterwards, the antibody was washed off and secondary antibodies were supplemented. Afterwards, the antibody was washed off and secondary antibodies were incubated. After reaction at room temperature for 1 hour, the slides were sealed and observed under microscope.

### 2.11. Statistical Analysis

Use SPSS 24.0 statistical software for *t*-test and one-way analysis of variance.

## 3. Results

### 3.1. Upregulated Expression of circSND1 in Tumor Tissues of Patients with Thyroid Cancer

**Figure 1:**
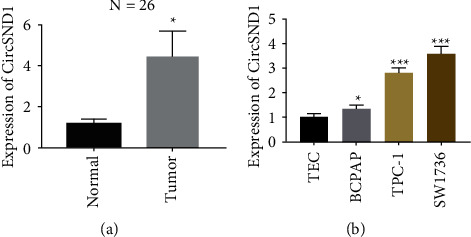
CircSND1 shows high expression pattern in thyroid carcinoma tissues and cells. (a) Analysis of the differential expressed level of circSND1 in thyroid carcinoma tissues and paracancer thyroid tissue. (b) Analysis of the differential expression of circSND1 in TEC cell line, BCPAP, TPC-1, and SW1736 cell lines. ^*∗*^*p* < 0.05, ^*∗∗∗*^*p* < 0.001.

The qRT-PCR technique was employed to monitor the quantification of circSND1 in thyroid cancer patients with matched cancer and adjacent tissues. The results uncovered that the expression of circSND1 in cancer was upregulated in contrast to that in corresponding paracancerous tissue (Figure 1(a)). Subsequently, we estimated the expressed levels of circSND1 in 3 different TC cell lines and uncovered that the quantification of circSND1 in BCPAP, TPC-1, and SW1736 cells was significantly higher than that in normal thyroid epidermal cells and TEC cell (Figure 1(b)). In addition, we can find that circSND1 has the highest expression in SW1736 cell, followed by TPC-1 cell, and finally BCPAP cell.

### 3.2. Knockdown of circSND1 Inhibits the Proliferative and Invasive Abilities of TPC-1 and SW1736 Cells

**Figure 2:**
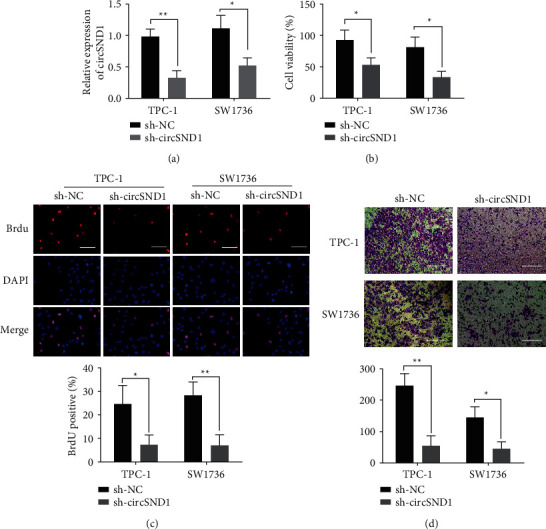
Downregulation of circSND1 restrained cell proliferative and invasive capacities of thyroid carcinoma cells. (a) Real-time fluorescent quantitative PCR was adopted to detect the expression of circSND1 in transfected TPC-1 and SW1736 cells of sh-circSND1 and sh-NC. (b) MTT method was employed to examine the cell viabilities of transfected TPC-1 and SW1736 cells of sh-circSND1 and sh-NC. (c) BrdU method was employed to examine the DNA synthetic abilities of transfected TPC-1 and SW1736 cells of sh-circSND1 and sh-NC. (d) Transwell method was employed to examine cell invasive abilities of transfected TPC-1 and SW1736 cells of sh-circSND1 and sh-NC. ^*∗*^*p* < 0.05, ^*∗∗*^*p* < 0.01.

We investigated the efficacy of circSND1 on the proliferative and invasive abilities of TC cells. First, circSND1 interference lentivirus was transfected into TPC-1 and SW1736 cells, and qRT-PCR was employed to monitor the quantification of circSND1. The results showed that, in contrast to negative control group (sh-NC), the expression of circSND1 in the circSND1-interfering lentivirus group (sh-circSND1) was radically reduced in TPC-1 and SW1736 cells, which was statistically significant (Figure 2(a)). After circSND1 interfering with lentivirus transfected TPC-1 and SW1736 cells, the MTT experiment indicated that, in TPC-1 and SW1736 cells, the cell viability of the circSND1 interference group was radically lower than that in control group (Figure 2(b)). The BrdU experiment indicated that, in TPC-1 and SW1736 cells, the DNA synthesis ability of the circSND1 interference group was significantly lower than that in sh-NC group (Figure 2(c)). And the transwell experiment indicated that, in TPC-1 and SW1736 cells, the invasion ability of the circSND1 interference group was radically lower than that in sh-NC group (Figure 2(d)).

### 3.3. Knockdown of circSND1 Inhibits Tumor Growth of Thyroid Cancer In Vitro

**Figure 3:**
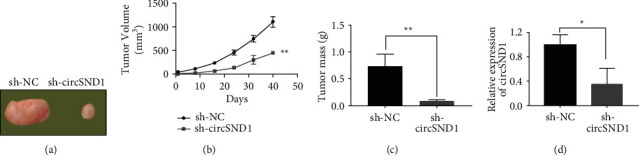
Knockdown of circSND1 inhibited tumor growth of thyroid cancer. (a) Representative pictures of tumors of sh-NC group and sh-circSND1 group. (b) Tumor volume of sh-NC group and sh-circSND1 group. (c) Tumor weight of sh-NC group and sh-circSND1. (d) circSND1 expression in tumor tissues of sh-NC group and sh-circSND1 group. ^*∗*^*p* < 0.05, ^*∗∗*^*p* < 0.01.

In order to confirm the influence of circSND1 on the progress of TC in vivo, TCP-1 cells were stably transfected with sh-circSND1 or a control vector (sh-NC) and injected subcutaneously into nude mice. In contrast with sh-NC group, the tumor size and weight in sh-circSND1 group were decreased significantly (Figures 3(a)∼3(c)). In addition, we uncovered that the quantitative level of circSND1 was decreased compared with sh-NC group (Figure 3(d)) validated by qRT-PCR.

### 3.4. MiR-182-5p Is a Direct Binding Target of circSND1

**Figure 4:**
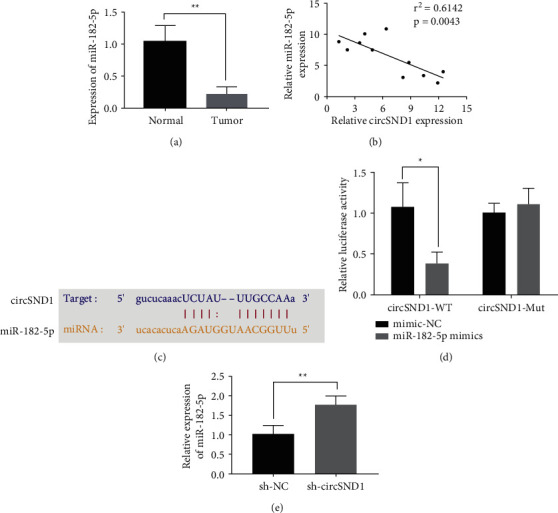
miR-182-5p is a direct binding target of circSND1. (a) Quantitative levels of miR-182-5p in TC tissues (tumor) and paracancer tissues (normal). (b) Detection of the correlation between miR-182-5p and circSND1 expression. (c) Prediction of the binding site of miR-182-5p and circSND1. (d) Luciferase assay was employed to examine the binding between miR-182-5p and circSND1. (e) Knockdown of circSND1 (sh-circSND1) promotes the expression of miR-182-5p compared with control (sh-NC). ^*∗*^*p* < 0.05, ^*∗∗*^*p* < 0.01.

We firstly tested the quantitative level of miR-182-5p in TC tissue; the results indicated that miR-182-5p quantification was radically lower in TC tissues (tumor) than that in normal group (Figure 4(a)). Pearson's correlation analysis suggested miR-182-5p expression was radically negatively related to circSND1 in TC tissues (Figure 4(b)). The starBase v3.0 [18] was employed to predict that miR-182-5p is a potential downstream target of circSND1 (Figure 4(c)). In order to confirm that circSND1 directly targets miR-182-5p, the wild-type or variant circSND1 sequence was inserted into the luciferase reporter gene plasmid. Subsequent luciferase reporter gene experiments uncovered that miR-182-5p can indeed bind directly to circSND1 (Figure 4(d)). In addition, we uncovered that downregulation of circSND1 promotes miR-182-5p quantification (Figure 4(e)). Taken together, these results show that circSND1 may act as a molecular sponge of miR-182-5p, thereby downregulating the quantitative level of miR-182-5p.

### 3.5. MET Is a Direct Binding Target of miR-182-5p

**Figure 5:**
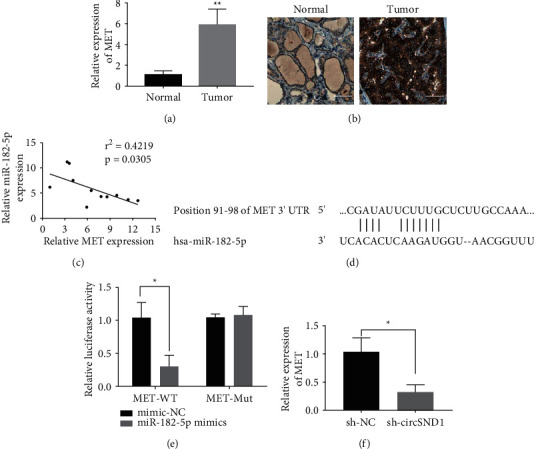
MET is a target gene of miR-182-5p. (a) qRT-PCR was adopted to examine the expression of MET in thyroid cancer tissues (tumor) and paracancer tissues (normal). (b) Immunohistochemical method was adopted to examine the expression intensities in TC tissues (tumor) and paracancer tissues (normal). (c) The linear correlations of MET and miR-182-5p expression were revealed by Pearson analysis. (d) Presumptive binding site of MET and miR-182-5p. (e) Luciferase assay was employed to examine the binding between MET and miR-182-5p. (f) Knockdown of circSND1 (sh-circSND1) inhibited the expression of MET compared with control (sh-NC). ^*∗*^*p* < 0.05, ^*∗∗*^*p* < 0.01.

For the purpose of testing the efficacy of MET on thyroid cancer, we confirmed its upregulation in TC tissues by qRT-PCR and immunohistochemical staining (Figures 5(a) and 5(b)). Next, Pearson's correlation analysis suggested MET expression was significantly negatively related to miR-182-5p (Figure 5(c)). The online software program TargetScan [19] was employed to predict that MET is a presumptive downstream target of miR-182-5p (Figure 5(d)). In order to confirm the hypothesis that miR-182-5p directly targets MET, the wild-type or variant MET sequence was inserted into the luciferase reporter gene plasmid. Subsequent luciferase reporter gene experiments uncovered that miR-182-5p can indeed bind directly to MET (Figure 5(e)). In addition, we disclosed that downregulation of circSND1 suppresses the quantitative level of MET (Figure 5(f)). We detected the changes of miR-182-5p and MET expression levels after circSND1 knockout in animal experiments. The results showed that miR-182-5p expression increased and MET expression decreased after circSND1 silencing (Figure S1). Taken together, the above results disclosed that miR-182-5p may directly bind to MET. The experimental results are consistent with the regulation mechanism of ceRNA.

### 3.6. CircSND1 Regulates MET Expression and Thyroid Cancer Cell Function via Binding miR-182-5p

**Figure 6:**
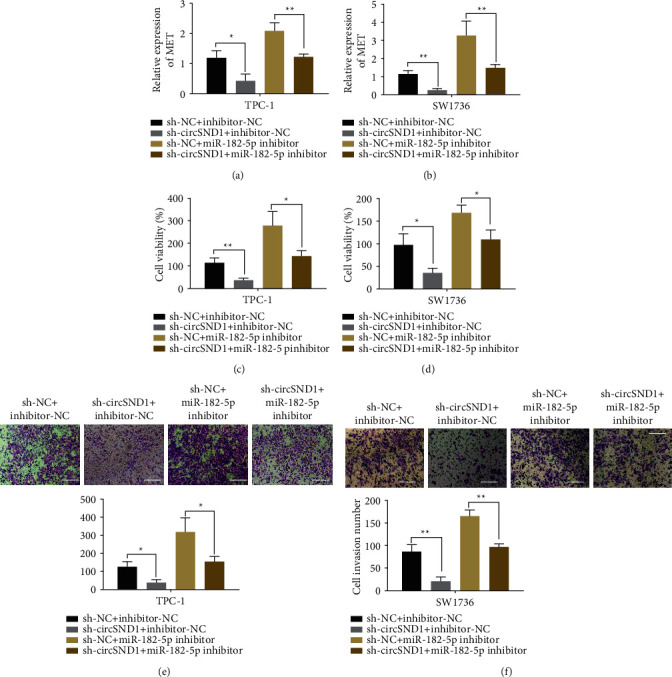
circSND1 regulated MET expression and thyroid cancer cell function via binding miR-182-5p. qRT-PCR was employed to examine the quantification of MET in TPC-1 cell (a) and SW1736 cell (b) transfected with sh-NC and inhibitor-NC, sh-circSND1 and inhibitor-NC, sh-NC and miR-182-5p inhibitor, or sh-circSND1 and miR-182-5p inhibitor. MTT method was employed to examine the cell viabilities of TPC-1 cell (c) and SW1736 cell (d) transfected with sh-NC and inhibitor-NC, sh-circSND1 and inhibitor-NC, sh-NC and miR-182-5p inhibitor, or sh-circSND1 and miR-182-5p inhibitor. Transwell assay was employed to examine the cell invasion abilities of TPC-1 cell (e) and SW1736 cell (f) transfected with sh-NC and inhibitor-NC, sh-circSND1 and inhibitor-NC, sh-NC and miR-182-5p inhibitor, or sh-circSND1 and miR-182-5p inhibitor. ^*∗*^*p* < 0.05, ^*∗∗*^*p* < 0.01.

To confirm that circSND1 regulates MET expression and TC cell function via binding miR-182-5p, we executed cotransfection experiments, constructed SW1736 and TPC-1 cells transfected with sh-NC + inhibitor-NC, sh-circSND1+inhibitor-NC, sh-NC + miR-182-5p inhibitor, and sh-circSND1+miR-182-5p inhibitor, respectively, and determined the quantitative level of MET, proliferation, and invasion through experiments. QRT-PCR results disclosed that MET relative quantification in SW1736 and TPC-1 cells increased notably after transfections of sh-NC + miR-182-5p inhibitor (Figures 6(a) and 6(b)). However, the upregulation of MET mediated by miR-182-5p inhibitor transfected was inhibited by sh-circSND1+miR-182-5p inhibitor cotransfection (Figures 6(a) and 6(b)). MTT experiments indicated that cell viabilities were obviously enhanced after transfections of sh-NC + miR-182-5p inhibitor in TPC-1 and SW1736 cells (Figures 6(c) and 6(d)). Transwell experiment indicated that cell invasion abilities were evidently enhanced after transfections of sh-NC + miR-182-5p inhibitor (Figures 6(e) and 6(f)). Therefore, we believed that circSND1 may compete with miR-182-5p to resist the inhibitory action of miR-182-5p on MET quantification.

## 4. Discussion

TC is one of the malignancies in adults, and its incidence is increasing with each passing year. A statistical figure based on the total number of cancers worldwide shows that the incidence of TC is a striking ninth in 2018 [20]. Papillary thyroid cancer (PTC) is the most frequent form of TC. The increased detection rate of TC, especially PTC, is considered to be the main reason for the increased incidence of TC, but fine needle aspiration biopsy and related mutation gene detection have certain limitations [21], and there are also controversies about the possible overdiagnosis and overtreatment of PTC. On the other hand, compared with other pathological types of TC, PTC is less malignant and has a better treatment effect and overall prognosis. However, 10% to 15% of patients may have distant metastasis, recurrence, or resistance to radioiodine therapy, leading to suffering and resulting in a decline in overall survival [22]. Although anaplastic TC (ATC) only represented 1% to 2% of TC, the death rate is as high as 20% to 50%, the median survival time is 3 to 6 months, the 1-year survival rate is less than 20%, and the 5-year survival rate is 0 to 5% [23, 24]. Therefore, looking for potential biomarkers to identify high-risk TC patients in the early stage of the disease and providing potential therapeutic targets are of strategic implication for the diagnosing, treating, and long-term management of TC sufferers. Literatures have uncovered that circRNAs are tightly linked to the progress of TC. CircRNA can participate in the regulation of genome transcription and translation processes at multiple expression levels, providing new and important targets for the clinical therapy of TC.

RNA is the direct bearer of hereditary code on chromosomes and plays a vital role in cell function. In recent years, more and more literatures have shown that the dysregulation of ncRNA is tightly linked to the pathogenesis of cancer, and the results of large-scale genome sequencing have also declared the key role of genetic changes in the progress of TC [25–27]. In the last few years, due to the emergence of deep sequencing technology, some RNAs without code capacity, such as microRNA (miRNA), lncRNAs, and circular RNA (circRNA), have been found to be abnormally expressed in thyroid cancer [28–31]. As a novel RNA without protein coding potential, circular RNA is still a hot spot in disease progresses, especially in tumors, in the last few years. CircRNA is highly conserved in sequence and has tissue-specific expression characteristics [32], which make circRNAs become potential molecular biomarkers of many diseases. CircRNA expression is disordered in thyroid cancer. For example, circRNA_RAPGEF5 is upregulated in TC tissues and cells and inhibits the expression of miRNA-198 and then upregulates the expression of the downstream target and fibroblast growth factor receptor 1 (FGFR1), which promote epithelial-mesenchymal transition (EMT), thereby enhancing cell proliferative and invasive capacities and inhibiting cell apoptosis [33]. CircRNA_0025033, located on chromosome 12, is increased in PTC tissues and cells and inhibits its target miRNAs, miRNA-1231, and miRNA-1304, thereby strengthening the DNA amplification and migration of TC cells and attenuating the apoptosis level [34]. In current study, ciscSND1 was upregulated in TC tissues and cells; however, its mechanisms are still unclear. Understanding the changes in specific circRNA expression profiles and the differences in biological functions in thyroid cancer is expected to be applied to early diagnosis, differential diagnosis, general survey of high-risk populations, tumor prognosis evaluation, and prediction of tumor metastasis and recurrence.

miRNAs are endogenous conservative RNAs without protein coding potential, which regulate gene expression and cell activity through negative feedback [35]. A variety of miRNAs are closely linked to TC progress, and their abnormal expression has been used as biomarkers in clinical applications to assist in the identification of TC as benign and malignant, the judgment of lymph node metastasis, recurrence monitoring, treatment, and prognosis prediction, such as miRNA-146 and miRNA -222 [36–39]. In ceRNA mechanisms, miRNA often acts as downstream target of circRNA. In our research, miR-182-5p may act as a binding target of circSND1. MiRNA-182 belongs to the miR-183/96/182 cluster and is expressed in multitudinous cells or tissues. Abnormal expression of miRNA-182 is involved in the progress of many diseases, including cancers. At present, researches on miR-182 regulating tumors are relatively in depth. For example, miR-182-5p is highly expressed in liver cancer tissues and cells and promotes the progression of liver cancer by inhibiting FOXO3a [40]. In addition, miR-182-5p also promotes the growth of oral squamous cell carcinoma via reducing CAMK2N1 quantification [41]. However, there are relatively few studies on miRNA-182 regulating thyroid cancer. In current study, miR-182-5p is a direct binding target of circSND1. Mesenchymal epidermal transformation factor (MET) is a presumptive therapeutic target for multitudinous cancers, including TC, and can regulate the proliferation of TC cells via PI3K/AKT signaling [42, 43]. Research shows that MET is a direct binding target of miR-34a in TC [44]. Many studies have confirmed that MET can promote tumor proliferation and metastasis. Xing et al. reported that activation of the c-MET pathway mobilizes an inflammatory network in the brain microenvironment to promote brain metastasis of breast cancer [45]. Cheng et al. reported that CXCR4 and c-MET cooperatively promote epithelial-mesenchymal transition in gastric cancer cells [46]. MET signaling affects cytoskeletal proteins such as paxillin, which participates in cell adhesion, growth, and motility [47]. In current research, we uncovered that MET is a downstream target gene of miR-182-5p, and its expression in TC tissues is significantly increased.

In conclusion, circSND1 was upregulated, while miR-182-5p was attenuated in TC. CircSND1 could expect to be regarded as miR-182-5p “sponge” and significantly contribute to TC tumorigenesis by activating MET expression. CircSND1 could be considered as the new molecular biomarker of TC tumor progression and diagnosis and provide novel insights for the treatment of TC.

## Data Availability

The data that support the findings of this study are available from the corresponding author upon reasonable request.

## Supplementary Materials

Supplementary Materials Figure S1: expression levels of miR-182-5p and MET in tumor tissues of nude mice in the control group and circSND1 silencing group.
