# Supplementary figures and images for: CircSND1/miR-182-5p Axis Promotes Proliferative and Invasive Abilities of Thyroid Cancer via Binding Targeting MET
Source: J Oncol. 2022 May 30;2022:9175084. doi: 10.1155/2022/9175084 (PMC9170435; doi:10.1155/2022/9175084)

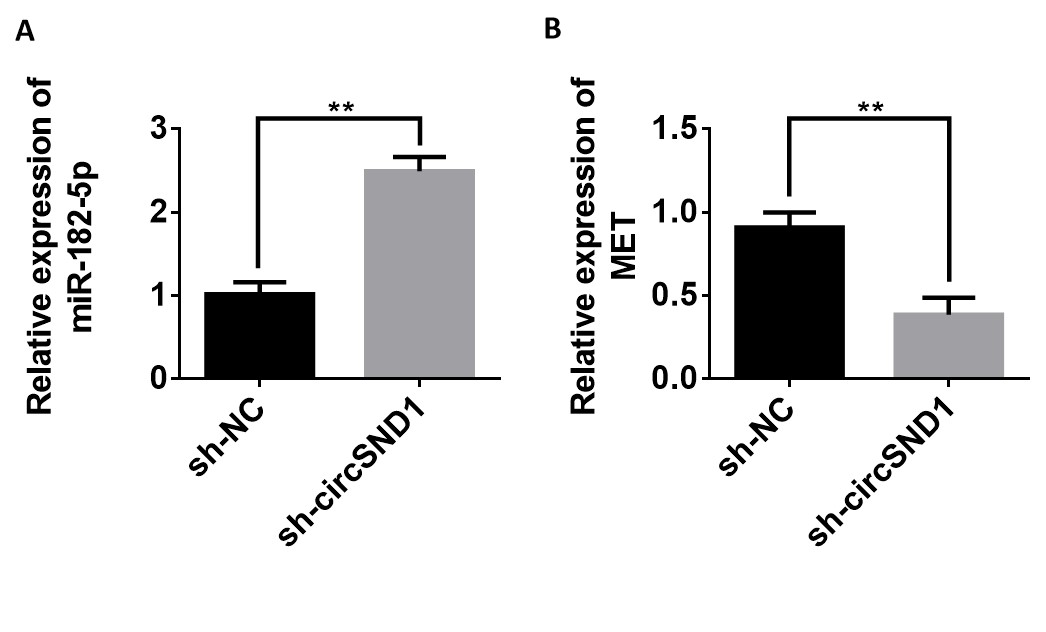

Supplement: Supplementary Materials — Figure S1: expression levels of miR-182-5p and MET in tumor tissues of nude mice in the control group and circSND1 silencing group. [file 9175084.f1.jpg]
